# Nature and climate change effects on economic growth: an LSTM experiment on renewable energy resources

**DOI:** 10.1007/s11356-021-13337-3

**Published:** 2021-03-29

**Authors:** Marco Mele, Antonia Rosa Gurrieri, Giovanna Morelli, Cosimo Magazzino

**Affiliations:** 1 University of Teramo, Teramo, Italy; 2 University of Foggia, Foggia, Italy; 3 Roma Tre University, Rome, Italy

**Keywords:** Renewable energy resources, Economic growth, Brazil, Sustainability, LSTM

## Abstract

Global energy demand increases overtime, especially in emerging market economies, producing potential negative environmental impacts, particularly on the long term, on nature and climate changes. Promoting renewables is a robust policy action in world energy-based economies. This study examines if an increase in renewables production has a positive effect on the Brazilian economy, partially offsetting the SARS-CoV2 outbreak recession. Using data on Brazilian economy, we test the contribution of renewables on the economy via a ML architecture (through a LSTM model). Empirical findings show that an ever-greater use of renewables may sustain the economic growth recovery, generating a better performing GDP acceleration vs. other energy variables.

## Introduction

Nowadays, several international organizations are warning about the negative socio-economic effects of the SARS-CoV2 health emergency, and its resurgence, that generated the most severe global economic recession since at least the 1930s, spreading more than 96 millions of people with two millions of deaths worldwide (WHO 2020). World economies are falling into an unprecedented deep freezing state, from which the recovery will be neither direct, nor automatic. Recently, the economic fallout and adverse effects of this pandemic have also hit Brazil, the largest South American economy, more than other North American and European countries. Therefore, also their policymakers have to implement a strategic medium-term plan to deal with this new double-side unknown macroeconomic shock.

At first glance, this new virus has had a general positive indirect impact on the environment in the temporary reduction of some greenhouse gas (GHG) emissions in the atmosphere because of the social distancing restriction policies adopted by the governments following the appearance of the pandemic. Although the decrease in GHG emissions currently observed in some countries is a positive result, environmental emergency remains. Decreasing GHG concentrations during a short period is not a sustainable way to clean up our environment. For a significant decline, there should be a long-term structural change in the major industrialized countries’ economies.

Global energy demand has continued to increase overtime, especially in emerging market economies, producing potential negative environmental impacts, particularly on the long term, on nature and climate changes. According to the agenda adopted by the UN General Assembly in 2015 on Sustainable Development Targets (UN 2015), Goal 7 recognizes the pivotal role of energy in social and economic development (Baruch-Mordo et al. 2019). It marks three aspects of energy access: ensure universal access to affordable, reliable and modern energy services, substantially increase the share of renewable energy, and double the global rate of improvement in energy efficiency. Nowadays, under SARS-CoV2 health emergency, affordable and reliable energy is critical for health facilities, since the pandemic has highlighted the need for consistent and stable electricity supply in health centers.

The world is making good progress on increasing access to electricity and improving energy efficiency. The global electrification rate rose, from 83% in 2010 to 90% by 2018. Latin America and the Caribbean and Eastern and South-Eastern Asia maintained strong progress, exceeding 98% access to electricity by 2018 (IEA 2020). However, the world energy balance is still largely dominated by fossil fuels, whose combustion accounted for 84% of global GHG emissions. A large-scale transformation of the global energy sector is possible, though it will require significant investments. For this reason, it is a shared goal of policy action to change worldwide the future energy matrix into a more sustainable and renewable sources scheme. The EU is committed to reach a target of at least 27% renewable energy of its overall energy consumption by 2030 (EC 2019).

Renewable energies are non-conventional energy sources constantly replaced by nature. They are grabbed from the sun, directly and indirectly, or from other natural features of the environment, and advantage environmental sustainability towards a more desiderable nature-climate equilibrium. They include solar energy, wind, falling water, the heat of the earth (geothermal), plant materials (biomass), waves, and ocean currents, temperature differences in the oceans and the energy of the tides, via technological applications, producing power, heat or mechanical energy by converting them, either to electricity or to transportation power. They will bring considerable benefits from a consumer perspective, from an environmental perspective, and from an economic perspective. Promoting renewables appear to be the one of the most efficient and effective solutions; they are the real fuel of the future, very attractive in world energy-based economies.

The gradual transition away from fossil fuels towards a carbon-neutral economy is one of the greatest challenges of our time. The case of Brazil is representative: it has a great potential for all renewable resources, via its geographical position that provides an excellent cycle of nature concerning climate issues. Therefore, it could use the environmental sustainability tool as an engine of economic recovery from SARS-CoV2, implementing a rapid process of structural changes, exploring a more intensive use of renewables, thus fostering GDP growth. In particular, enhancing investments in this sector, Brazil could mitigate the effects of the domestic and international economic crisis caused by COVID-19. It has abundant resources of solar, wind, biomass, and ocean energy, and is the third largest energy market in the world for renewables, after China and the United States, with very ambitious goals in this sector for the future. These conditions make it possible to switch in the long term to renewable features that differentiate the Brazilian energy matrix from the world one. Its extensive endowment of natural resources allows the realization of sustainable projects able to support the growing electricity demand, enforcing economic development. Appropriate transmission networks will encourage producing, delivering and consuming energy, efficiently connecting new production centers to the Brazilian megalopolises.

Furthermore, as the demand for control of GHG emissions is getting stronger all over the world, Brazil might also consolidate its potential as a great exporter, with benefits for its GDP rate of growth; the development of interconnections within Brazil and neighboring countries might generate a system capable of responding to the challenges deriving from renewable energies. This condition will support a new ruling in the Latin American electric sector via the creation of a “Single Latin American Electricity Market”. Smart distribution networks will be crucial for the management of the energy flows through digital platforms, growing penetration of electricity in end uses, distributed generation, renewable sources, electric mobility, and the need for ever-higher quality of service.

Several countries question about the role they can play in stopping the deterioration of natural resources that undermine economic activity. Brazil have had a significant role in the process, contributing for 44.8% of clean energy since 2010, fast moving to a green growth using renewables, an important part of its primary energy demand. Promoting renewable energy policy measures are, therefore, one of the biggest challenges for this country. The Northeast region is the primary producer of wind energy. With 135 parks, Rio Grande do Norte is the state where most of the energies produced use the force of the winds, with 3678.85 MW of installed capacity. Thus, Brazil’s rising energy-related GHG emissions and investing in renewable energy policy might have a positive return to the economy, mitigating carbon emissions and making energy sector one of the least carbon-intensive in the world.

Economic literature has analyzed the nexus between economic growth and environmental degradation starting from the so-called Environmental Kuznets Curve (EKC), which shows a positive correlation between environmental degradation and economic growth to a certain level, beyond which an increase in the quality of environment allows a per capita income growth (Churchill et al. 2018). Based on the Intergovernmental Panel on Climate Change reports (IPCC 2014), the renewables could meet, by 2050, 77% of the world energy needs, with respect to the current relatively low value (13%).

Fossil fuels are still the primary sources of global energy, covering over 80% of the total power supplied in the world economy. A higher consumption of them leads to higher GHG emissions, particularly carbon dioxide (CO_2_), which contribute to global warming. However, their use across the globe faced several obstacles in many countries, including Brazil, pushing them to find alternative sustainable energy sources. Among these challenges, there are the increasing disconnection between the demand of energy and its supply in the global market, the growing depletion of oil reserves, and emissions of harmful gases in the atmosphere.

Carbon, a byproduct of the combustion of fossil fuels, is the leading cause of the current ecological and environmental crisis. Concerning the various catastrophes that have shocked the energy sector, lately Brazil has displayed an increasing interest to develop the production of clean and renewable energy sources. Besides the depletion of fossil fuel, another factor that has prompted the Brazilian government to embrace a “Green Growth Agenda” is the persistent rate of environmental degradation, fostering for tandem coexistence of economic growth and ecological conservation (Inglesi-Lotz 2016).

This study aims to verify, in times of COVID-19, the possibility that a more intensive use of renewable energy (and therefore more significant investments in this field) could generate an acceleration of Brazil’s GDP. We will verify this hypothesis in an historical context, from 1990 to 2018, using a machine learning (ML) approach.

The paper proceeds as follows. The “Literature review” section presents the essential literature review. The “Data and methodology” section shows the data and empirical methodology. The “Results” section comments the empirical findings. Finally, the “Conclusions and policy implications” section concludes and gives some policy implications.

## Literature review

According to Vaona (2012), renewable energy resources are highly suitable in reducing the amount of carbon in energy, which is an essential element of the climate mitigation process. Following Oliveira and Trindade (2018), the consumption of renewable energy would minimize the emission of carbon by approximately 8.2% by 2050. The use of clean energy technologies enormously contributes also to push up the economic development of the area, minimizing the dependency on imported fuels. It increases the consumption and accessibility of energy across Brazil to over 1.4 million people who are not fully exposed to renewable energy sources (Newman 2017). The spread of renewable energy can also create new job opportunities and facilitate industries growth in underdeveloped areas. Menyah and Wolde-Rufael (2010) showed that an increase in energy consumption by 1% encourage a multiplier effect of 0.12% on Brazil’s per capita GDP.

Lately, there is a growing interest in investigating the one-way nexus between economic growth and clean energy demand. Many studies exhibited a positive causality between the consumption of renewable energy resources and the Brazil GDP growth rate. Inglesi-Lotz (2016) used Pedroni co-integration techniques on a 34 countries sample; Omri et al. (2015) a dynamic data model on a panel of simultaneous equations on 17 developed countries; Newman (2017) a Vector Error Correction model to analyze the Brazilian economy from 1980 to 2010. Others, instead, using different econometric approaches, claim the presence of a negative correlation between the two variables. In particular, Inglesi-Lotz (2016) stated an inconsistent result, estimating a negative correlation between renewables and growth. These findings were also corroborated by Oliveira and Trindade (2018), showing that the Energy Returns on Investments for recycled energy is much lower than on technology; hence, clean energy sources are highly unlikely to be the primary sources of power in the future due to such a substantial disparity. They noted that there is no strong correlation between the production of renewable energy and the rate of growth of a country.

Further, Menegaki (2011), using the Granger causality test on EU data (1997–2007), shows the non-existent causality between GDP and the consumption of renewable energies, because of the early stages of development and the market penetration of clean energy across Europe. In addition, still applying the Granger causality tests, Vaona (2012) confirms the existence of the neutrality hypothesis in connection to the consumption of renewable energy and the economic growth of Brazil during 1861–2001. In conclusion, there is no clear evidence of a robust correlation between renewable energy consumption and economic growth in Brazil; economic literature rather indicates mixed results.

Brazil is one of the very few countries that have fully utilized renewable energy sources in the world. Different factors prompted the attention to the generation and use of renewable energy. Among them are the growing part of the population that still cannot be fully supplied with hydropower, the depletion of fossil fuel, and the increasing number of motor vehicles, and other equipment, which requires energy for operations. In the face of an increase in energy consumption in Brazil, many researchers are still trying to prove the existence of a long-term causal relationship on the usage of renewable energy sources and the related change in terms of growth.

## Data and methodology

In order to test if a more intensive use of renewable energy (and therefore more significant investments in this field) could generate a significant acceleration on the Brazilian economy, we use a ML approach based on long short-term memory network (LSTM), following Magazzino et al. (2020a, 2020b, 2021), and Mele and Magazzino (2021). Unlike traditional regression methods, ML is non-parametric techniques. Besides, they are not bound by a functional form but are free from the assumption of statistical distribution. In parametric techniques, such as linear regression, an increase in the independent variable determines only a variation, positive or negative, on the dependent variable. In ML techniques, on the other hand, the effect of the independent variable on the dependent one can produce a different result due to the levels and interactions with other variables. The ability to capture these interactions through different values of variables allows these techniques to go beyond traditional parametric models. With this approach, we estimate, in an historical context from 1980 to 2018, the acceleration probability of Brazilian GDP (dGDP) with a larger use of renewable energy consumption (Renewable energy consumption). We use a combination of four variables (Table 1) from 1980 to 2018.

The algorithm construction model, predicting the acceleration between the target (dGDP) and the three inputs, is based on a complex mathematical function. The starting mathematical formulation is as follows:

1 $$ {h}_{\theta }\ \left({x}_1\right)={\theta}_0+{\theta}_1{x}_1\ \mathrm{where}\ \theta =\left\{\begin{array}{c}{\theta}_1\\ {}{\theta}_2\end{array}\right.,x=\left\{\begin{array}{c}1\\ {}{x}_1\end{array}\right. or\ {h}_{\theta }\ (x)={\theta}^tx $$

Now we solve the minimization problem with the cost function:

2 $$ J\left({\theta}_0,{\theta}_1\right)=\frac{1}{2m}\ \sum \limits_{i=1}^m{\left({h}_{\theta}\left({x}^{(i)}\right)-{y}^{(i)}\right)}^2\ \mathrm{where}\ {\displaystyle \begin{array}{c}\mathit{\min}\\ {}\left(\theta 0,\theta 1\right)\ \end{array}}J\left({\theta}_0,{\theta}_1\right) $$

where:

3 $$ {\theta}_j\leftarrow {\theta}_j\hbox{--} \alpha\ \frac{\delta }{\delta\ \theta j}J\ \left(\theta \right) $$

Utilizing the Cauchy approach, we identify:

i. *θ*_*0*_ and *θ*_*1*_ with two initial values, chosen randomly;
ii. two new parameters *θ*_*1*_ and *θ*_*2*_ in such a way as to reduce the value calculated by *J(θ*_*0*_*, θ*_*1*_*)*;
iii. reach the minimum of the function.

4 $$ {\theta}_j={\theta}_j\hbox{--} \alpha\ \frac{\delta }{\delta\ {\theta}_j}J\ \left({\theta}_0\hbox{--} {\theta}_1\right) $$

where *α* is learning rate and serves to establish when the displacement to be made along the function must be wide.

Now, consider the following basic assumptions about a neural network:

1. *x* ∈ *ℝ*^*D*^ with output vector *y* ∈ *ℝ*^*k*^
2. layer-wise output vector *ol* ∈ *ℝ*^*M*^, to each layer *l = (1,2…, L)*
3. there will always be a matrix *W*^*l*^ ∈ *ℝ*^*Mt* × *Mt* − 1^ with layer *l = (1,2…,L)*, *→M*_*0*_ *=D* and *M*_*L*_ *= K*
4. vector *b*^*l*^ ∈ *ℝ*^*Mt*^, layer *l = (1,2…,L)*
5. *θ* will be the set of parameters
6. *σ* (·) : *ℝ* → *ℝ* will be a non-linear derivable scalar function, and his derivate will be *σ*^*1*^.

Our feed-forward neural network (FFNR) in LSTM is:

5 $$ f\left(x,\theta \right)={o}^L=\to {o}^l=\sigma \left({a}^l\right)=\sigma\ \left({W}^l{o}^{l- 1}+{b}^l\right),\mathrm{with}\ l=\left[ 1, 2\dots, L\right]\ \mathrm{and}\ {a}^l={W}^l{o}^{l- 1}+{b}^l\ {o}^0=x $$

We decide now to choose a two-layer network: *L = (2)* with *b*^*l*^ *= 0*.

Our functions become:

6 $$ f\left(x,\theta \right)={o}^2=\sigma\ \left({W}^2{o}^1\right)=\sigma\ \left({W}^2\ \sigma\ \left({W}^1x\right)\right) $$

If *σ* is not linear:

$\sigma (x)=\frac{1}{1+\mathit{\exp}\left(-x\right)};\sigma^{\prime }(x)=\sigma\ (x)\ \left(1-\sigma\ (x)\right)\to$ if the output is in the range (−1;1) there is a tanh function:

$\mathit{\tanh}\ (x)=\frac{e\uparrow x-e\uparrow -x}{e\uparrow x+e\uparrow -x};\mathit{\tanh}^{\prime }(x)=1\hbox{--} {\mathit{\tanh}}^2\ (x)\to$ with rectified linear unit:

7 $$ Relu(x)=\left\{\begin{array}{c}x;x>0\\ {}0;x\le 0\end{array}\right. $$

8 $$ MSE\ \left(\theta \right)=\frac{1}{2N}\ \sum \limits_{n=1}^N II\ f\ \left({x}_n,\theta \right)\hbox{--} {t}_n\  II{\displaystyle \begin{array}{c}2\\ {}2\end{array}} $$

$$ f\left({X}_n,\theta \right)\hbox{--} {t}_n\  II{\displaystyle \begin{array}{c}2\\ {}2\end{array}} $$ will be the squared about the normal Gaussian.

To calculate the backpropagation effect in the model, we use:

9 $$ \frac{\mathrm{\partial E}}{\partial {W}_{ij}^l}=\frac{1}{N}\ \sum \limits_{n=1}^N\left(f\ \left({\mathrm{x}}_n,\theta \right)\hbox{--} {\mathrm{t}}_{\mathrm{i}}\right)\ \frac{\partial }{\partial {W}_{ij}^l}f\ \left({\mathrm{x}}_{\mathrm{i}},\theta \right) $$

We calculate the chain rule of derivates for

$$ \frac{\partial }{\partial {W}_{ij}^l}f\ \left({\mathrm{x}}_{\mathrm{i}},\theta \right) $$: (10)

$$ \frac{\partial }{\partial {W}_{ij}^l}f\ \left({\mathrm{x}}_{\mathrm{i}},\theta \right)=\frac{\partial }{\partial {W}_{ij}^l}{\mathrm{o}}^L=\frac{\partial }{\partial {W}_{ij}^l}\left[\upsigma\ \left({\mathrm{a}}^{\mathrm{L}}\right)={\upsigma}^{\prime}\left({\mathrm{a}}^{\mathrm{L}}\right)\frac{\partial }{\partial {W}_{ij}^l}\left({\mathrm{a}}^{\mathrm{L}}\right)={\upsigma}^{\prime}\left({\mathrm{a}}^{\mathrm{L}}\right)\frac{\partial }{\partial {W}_{ij}^l}\left({\mathrm{W}}^{\mathrm{L}}{\mathrm{o}}^{\mathrm{L}}+{\mathrm{b}}^{\mathrm{L}}\right)={\upsigma}^{\prime}\left({\mathrm{a}}^{\mathrm{L}}\right){\mathrm{W}}^{\mathrm{L}}\ \frac{\partial }{\partial {W}_{ij}^l}\ {\mathrm{o}}^{\mathrm{L}-1}={\upsigma}^{\prime}\left({\mathrm{a}}^{\mathrm{L}}\right){\mathrm{W}}^{\mathrm{L}}\ \frac{\partial }{\partial {W}_{ij}^l}\ \upsigma \left({\mathrm{a}}^{\mathrm{L}-1}\right)=\upsigma^{\prime}\left({\mathrm{a}}^{\mathrm{L}}\right){\mathrm{W}}^L\upsigma^{\prime}\right({\mathrm{a}}^{\mathrm{L}-1\Big)}{\mathrm{W}}^{\mathrm{L}-1}\ \frac{\partial }{\partial {W}_{ij}^l}\ {\mathrm{o}}^{\mathrm{L}-2}=a^{\prime}\left({a}^L\right)\ {W}^L\ \sigma^{\prime}\left({a}^{L-1}\right)\ {W}^{L-1}\dots \frac{\partial }{\partial {W}_{ij}^l}\ {o}^l=\prod \limits_{K=l+1}^L\left(\sigma^{\prime}\left({a}^k\right)\ {W}^k\right)\ \frac{\partial }{\partial {W}_{ij}^l}\ {o}^l\frac{\partial }{\partial {W}_{ij}^l}{\mathrm{o}}^1=\frac{\partial }{\partial {W}_{ij}^l}{\mathrm{o}}^{\mathrm{l}}\left[{\upsigma}^{\prime}\left({\mathrm{W}}^{\mathrm{l}}{\mathrm{o}}^{\mathrm{l}-1}+{\mathrm{b}}^{\mathrm{l}}\right)\right]={\upsigma}^{\prime}\left({\mathrm{a}}^{\mathrm{l}}\right)\frac{\partial }{\partial {W}_{ij}^l}\left({\mathrm{W}}^{\mathrm{l}}\ {\mathrm{o}}^{\mathrm{l}-1}+{\mathrm{b}}^{\mathrm{l}}\right)=\upsigma '\left({\mathrm{a}}^{\mathrm{l}}\right)\ {o}_j^{l-1} $$

## Results

The results confirm the goodness of the model in predictive events (Table 2). In fact, our algorithm lies within a ROC curve with values greater than 85%. Therefore, the model neither has false positives, nor negatives.

**Table 1:** List of variables

dGDP	Per capita GDP in 1990 US $, converted at Geary–Khamis PPPs	Total Economy Database (TED)
Energy use	Per capita energy consumption (kg of oil equivalent)	International Energy Agency (IEA)
Renewable energy consumption	Renewable energy consumption (% of total final energy consumption)	International Energy Agency (IEA)
Combustible renewables and waste	Combustible renewables and waste (% of total energy)	World Bank

**Table 2:** AUROC results

LSTM	dGDP	Energy use	Renewable energy consumption	Combustible renewables and waste
Training set	0.91851	0.87652	0.84562	0.86154
Test set	0.85622	0.66552	0.87818	0.73251

**Table 3:** Training set AUROC on dGDP

Variables	ITE 1	ITE 2	ITE 3	ITE 4
Energy use	0.79261	0.80991	0.80952	0.80095
Renewable energy consumption	0.79246	0.80616	0.83661	0.83994
Combustible renewables and waste	0.65678	0.74915	0.78441	0.77441

**Table 4:** Test set AUROC on dGdp

Variables	ITE 1	ITE 2	ITE 3	ITE 4
Energy use	0.78495	0.79523	0.79554	0.80029
Renewable energy consumption	0.79541	0.79985	0.81547	0.81597
Combustible renewables and waste	0.61411	0.71019	0.71952	0.71998

**Fig. 1:**
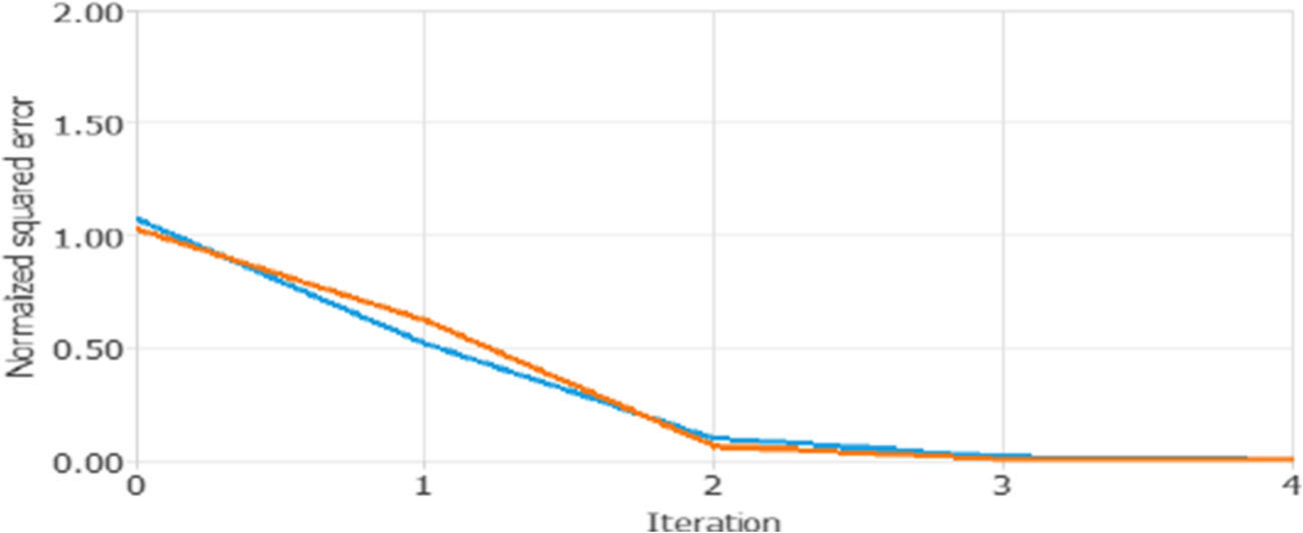
Levenberg-Marquardt algorithm errors history (dGDP). Source: Our elaborations in Oryx 2.0.8

Next, we generated in the algorithm the ability to predict, concerning the entire historical dataset (1980–2018), the variation of the Target dGDP in four ITEs. Through this procedure, we analyze the inputs effects on interconnected networks over time. Since our dataset is an annual time series, the four ITEs could be interpreted as the four-year period after 2018. In other words, we try to predict if, one or more inputs, can determine the variation of the Target (dGDP) until 2022. This process is performed through the Levenberg-Marquardt algorithm errors history (Fig. 1).

The results of the test (Fig. 1) are significant since the algorithm that foresees different variations over time (four ITEs) shows that the squared errors by the predictions of each single ITE decrease up to the minimal minimum.

The results in Tables 3 and 4 represent the effect over time of three inputs (Energy use, Renewable energy consumption, Combustible renewables and waste) on the target variation (dGDP). The algorithm processed 86,947 redundancy combinations between the inputs and the target in an unsupervised system. We limited the predictive cycles that recorded similar values for at least three times for each cycle through the Sunburst test. With this system, the model was purified from the “chain repeat” effect. Subsequently, for each ITE, we recorded the values in logarithmic and variation scale: this system allowed us to analyze multiple data contexts whose redundancy combination recorded a maximum value of 347,788 combinations in ITE4.

In the algorithm, all our inputs show positive effect as the time iterations turn. Both, in the Training and the Test set, the Renewable energy consumption variable had better perform the target than the other inputs. In particular, Energy use (more generally) has a positive effect on GDP, but lower than the one reached by renewables. The rationale for this result is in the projections concerning the four ITEs based on the assumption of no friction on the labor market following Brazil transition process to a larger economic sustainability program. In particular, the workforce will adapt both to structural changes and to skills requirements. This scenario designs a positive effect not only on nature and the climate change but, also, on potential GDP growth and employment rate as the result of the investment activities necessary to enhance this transition in the medium-term, together with a positive impact on fossil fuel imports.

The shift towards the production of capital goods, such as renewable energy equipment and machinery, will lead to a significant increase in the labor demand from related economic activities.

However, if this acceleration towards energy transition will increase GDP, and jobs globally, the advantages are not automatic. Some communities could suffer from it, especially if they are heavily dependent on fossil fuels production for local welfare. Higher investments in education, in human capital formation, and in social security measures are necessary and suggested policy actions. The increase in per capita GDP will be able to satisfy the energy needs of a growing population, to enforce development of the entire area, while reducing GHG emissions and increasing the productivity of natural resources.

Our empirical findings provide robust evidence that nature preservation and economic growth are compatibles targets, dispelling the doubts of the traditional economic theory on the unsustainability of the nexus between GDP and exploitation of natural resources. Besides, the acceleration effect of the input of renewable energies is, therefore, the best choice for policymakers in a period of economic uncertainty as SARS-CoV2 outbreak recession.

## Conclusions and policy implications

In the last decade, Brazil has experienced a rapid growth towards the production of renewable resources in the domestic energy sector. These sources are one of the crucial drivers of the Brazilian economy recovery after the government shifted its effort into a full utilization of clean energy. The energy policies measure up well against the world’s most urgent energy challenges. Access to electricity across the country is almost universal and renewables meet almost 45% of primary energy demand, making Brazil’s energy sector one of the least carbon-intensive in the world.

Several researchers are confident of a positive correlation between economic development and renewable energy consumption worldwide. In this context, Brazil fits well, since it is one of the largest worldwide energy consumers and producers. However, the economic growth-renewable energy resources nexus could reverse, as effect of the domestic and international economic crisis from COVID-19. The pandemic and resulting fall in demand for energy is hitting the willingness to fund oil and gas projects more than those involving renewable energies. Therefore, economic policy solutions are needed. In particular, they should be based on higher investments in renewables to accelerate the Brazilian long-term development process.

The economic literature has not yet fully addressed either the sign of the nexus between renewable energy consumption and economic growth, or the effective benefits from clean energy production around the world. Our work shows that a greater use of renewable energies could foster economic growth. Through an LSTM model, we verified how an increase in renewable energy consumption triggers an acceleration of the Brazilian GDP with respect to other energy sources. Compared to other standard econometric models, our study automatically captured GDP per capita as best Target (output). A positive variation of it, through a predictive process of 4 ITEs, is due to an acceleration of renewable energies.

Therefore, in times of international pandemic, Brazil should offset the negative effects that will spill over the economic system. In this country, where every President changes previous energy policies, calculating the policy output index (Pischke et al. 2019), might helpful for policymakers. Using defective policies, they should intensify the country energy structural change process by promoting a more intensive use of renewable energy resources.

## Data Availability

If requested, we are available to disseminate the data of the paper.
